# Health services during Covid-19: What do we know of the degree of service disruption and size of the backlogs?

**DOI:** 10.1093/eurpub/ckac129.210

**Published:** 2022-10-25

**Authors:** F Tille, T Zapata

**Affiliations:** 1 London Hub, European Observatory on Health Systems and Policies, London, UK; 2 Health Workforce and Service Delivery, WHO Regional Office for Europe, Copenhagen, Denmark

## Abstract

Since the onset of the COVID-19 pandemic, there have been concerns that shifting health system capacities towards acute COVID-19 cases can affect the provision of non-COVID-19 essential health services, causing severe disruptions and lack of care. Examples of this have been seen during other epidemic outbreaks, such as the 2014-2016 Ebola outbreak in West Africa. To capture the degree of service disruption across the European and Central Asian region, we analysed data from the World Health Organization's Pulse Survey on the Continuity of Essential Health Services, conducted in three rounds in 2020 and 2021. The key findings include:

– Health service provision has been heavily disrupted in virtually all countries. 91% of countries reported service disruptions in late 2021, indicating that health services continue to be disrupted at large scale.

– Service discontinuation has been substantial across all levels of care and in most service areas, often resulting in delays and cancellations of elective and emergency procedures, routine visits, prescription renewals, and referrals to specialty care. This has led to growing backlogs and record waiting times for services.

– Countries have been affected to varying degrees and report different levels of service disruption, size of the backlog, recovery of services aiming for pre-pandemic levels, and interventions to manage waiting lists.

The findings indicate that even as health systems are better learning to care for acute COVID-19 patients, the pandemic's impact on essential health services is massive and likely to affect the care for people's health and well-being post the acute phase of the public health emergency. Measuring the size of backlogs and implementing innovative care solutions are urgent and paramount.

